# Elemental and Molecular Segregation in Oil Paintings due to Lead Soap Degradation

**DOI:** 10.1038/s41598-017-11525-1

**Published:** 2017-09-14

**Authors:** Yu-chen Karen Chen-Wiegart, Jaclyn Catalano, Garth J. Williams, Anna Murphy, Yao Yao, Nicholas Zumbulyadis, Silvia A. Centeno, Cecil Dybowski, Juergen Thieme

**Affiliations:** 1 Department of Materials Science and Chemical Engineering, Stony Brook University, Stony Brook, New York, USA; 2 National Synchrotron Light Source II, Brookhaven National Laboratory, Upton, New York, USA; 3 Department of Chemistry and Biochemistry, Montclair State University, Montclair, New Jersey, USA; 4 Department of Chemistry and Biochemistry, University of Delaware, Newark, Delaware USA; 5 Independent Researcher, Rochester, New York, New York, USA; 6 Department of Scientific Research, The Metropolitan Museum of Art, New York, USA

## Abstract

The formation of Pb, Zn, and Cu carboxylates (soaps) has caused visible deterioration in hundreds of oil paintings dating from the 15th century to the present. Through transport phenomena not yet understood, free fatty acids in the oil binding medium migrate through the paint and react with pigments containing heavy metals to form soaps. To investigate the complex correlation among the elemental segregation, types of chemical compounds formed, and possible mechanisms of the reactions, a paint sample cross-section from a 15th century oil painting was examined by synchrotron X-ray techniques. X-ray fluorescence (XRF) microscopy, quantified with elemental correlation density distribution, showed Pb and Sn segregation in the soap-affected areas. X-ray absorption near edge structure (XANES) around the Pb-L3 absorption edge showed that Pb pigments and Pb soaps can be distinguished while micro-XANES gave further information on the chemical heterogeneity in the paint film. The advantages and limitations of these synchrotron-based techniques are discussed and compared to those of methods routinely used to analyze paint samples. The results presented set the stage for improving the information extracted from samples removed from works of art and for correlating observations in model paint samples to those in the naturally aged samples, to shed light onto the mechanism of soap formation.

## Introduction

Heavy metal soap formation is a major issue for the conservation of paintings and other objects of cultural significance bearing oil-based media and pigments composed of heavy metals^1–9^. In the case of oil paintings, the saponification process typically involves heavy metals such as Zn, Pb, and Cu that react with the free fatty acids in the oil binding medium, causing different forms of deterioration. In old master paintings, Pb soaps are the most frequently observed, due to the widespread use by artists of the lead-containing pigments lead white, 2PbCO_3_·Pb(OH)_2_, and lead tin yellow type I, Pb_2_SnO_4_. Lead soaps form protrusions up to 200 microns in diameter that may break through the paint surface, as in the case of *Madame X*, by J. S. Sargent. Paint layers may become transparent and make the support visible, such as in Meindert Hobbema’s *Village among Trees*. Soaps may also lead to the development of surface crusts, as observed in *Portrait of a Woman* by Jan van Ravesteyn, and in other 17^th^ century Dutch paintings^5^.

Soap formation has been identified in hundreds of works of art dating from the 15^th^ century to the present, and in numerous cases it has been linked to environmental factors such as light exposure, changes in relative humidity, and/or elevated temperatures^10^. However what factors trigger the process, what the mechanisms are, and how it can be arrested or prevented is not well understood^5^. Therefore, knowledge of the chemistry of the process is crucial to help preserve the affected works of art.

A wide range of methods have been used to characterize soap deterioration in paintings and to study the reactions in model paint samples. Spatially resolved techniques such as Raman spectroscopy, Fourier-transform infrared spectroscopy (FTIR), scanning electron microscopy - energy dispersive X-Ray spectroscopy (SEM-EDS), secondary ion mass spectrometry (SIMS), and XRF have been employed to analyze the soaps localized in paint cross-sections and, along with Gas chromatography–mass spectrometry (GC-MS) and direct temperature-resolved mass spectrometry (DTMS), in sample scrapings and model paint samples^1–6,10–21^. Raman spectroscopy and FTIR instrumentation used in laboratories generally allow one to obtain molecular information with spatial resolutions on the order of a few microns; SEM-EDS gives elemental mapping with a spatial resolution of ~ a micron and SIMS has a spatial resolution in the order of 100 nanometers^15^. Of these techniques, only SIMS, that involves sputtering the sample during the measurements, permits one to obtain elemental *and* molecular information in the same setting. The need of a wide range of techniques to characterize the saponification process reflects the complexity of the questions associated with this deterioration process.

Solid-state nuclear magnetic resonance (NMR) was successfully applied to determine the structure of a series of Pb carboxylates^22,23^ and to study the reaction between PbSn yellow type I and palmitic acid in model paint samples^24^. Powder and single X-ray diffraction patterns have also been reported for Pb, Zn, and Cu soaps^11,23,25^. XRF mapping and confocal micro-XRF (μ-XRF) have been shown to be particularly useful for analyzing the elemental distribution in Pb soap protrusions^4,26^. Faubel *et al*. utilized confocal μ-XRF to show the elemental distribution of Ba, S, Ca, Pb, and Zn in the paint, while focusing on the role of Zn in the protrusions^26^. The use of a micron-sized beam in XRF mapping, when combined with scans of the X-ray photon energy, allows micro-X-ray absorption near-edge structure (μ-XANES) spectroscopy across the Pb-M_4_ & M_5_ edges to differentiate Pb pigments and Pb soaps, as well as to obtain the elemental distributions of Pb, Si, Br, and Fe^4^.

The elemental segregation in a paint sample containing PbSn yellow and soaps was previously examined using SEM-EDS by Keune and Boon^12^. In the present work, results of the study of the soaps formed in a 15th century oil painting, *The Crucifixion* by Jan Van Eyck^27,28^, show that the combination of synchrotron XRF microscopy and μ-XANES, both with a spatial resolution in the order of a micron, permits one to determine the elemental and molecular distributions thus providing complementary information to other techniques routinely used in conservation science laboratories. The ability to carry out elemental *and* molecular analysis in the same setting is beneficial. The hard X-ray capabilities available at the Sub-micron Resolution X-ray Spectroscopy (SRX) beamline are a promising alternative to probe Pb compounds at the L_3_ edge of Pb, allowing the use of higher X-ray energies in comparison to studies with M edges used previously^4^. For more complex samples, such as for studying features that are below the surface of specimens or for *in situ* experiments, X-rays also provide the possibility to work at different depths and, therefore, to gather bulk information that is not accessible by surface-sensitive techniques. However, one should pay attention to potential self-absorption effect if X-ray fluorescence measurements are conducted; the limitations of the techniques regarding this and other aspects are also discussed and summarized in the conclusion.

## Experiments

### Sample preparation and light microscopy characterization

The Pb-containing pigments Pb white and PbSn yellow type I were purchased from Sigma-Aldrich and Kremer Pigments Inc., respectively. The Pb azelate and Pb palmitate soaps were synthetized by methods adapted from previously published protocols^7,11^. Previous studies on similar pigment samples using NMR show that Pb white contains a 5% lead carbonate impurity^29^ and that PbSn yellow type I contains the following impurities: 1.83 ± 2 mole % Pb_2_SnO_4_, 5.2 ± 1.1 mole % Pb_3_O_4_, and 11.8 ± 0.9 mole% SnO_2_^24^. Our sample, a paint chip removed from the original frame in Jan Van Eyck’s *The Crucifixion* (MMA #37.92a), *ca*. 1440–1441, was mounted as a cross-section using a Technovit^®^ resin and was initially characterized by light microscopy and Raman spectroscopy.

### Raman spectroscopy

Raman spectra were recorded with a Renishaw System 1000 spectrometer, using a 785 nm laser. The laser beam was focused on different spots in the sample cross-section using a 50× objective lens allowing a spatial resolution of 2–3 μm. Powers of 1–5 mW were used, with accumulation times of 40 seconds. The spectra were recorded using a 1200 lines mm^−1^ grating and a CCD detector, allowing a spectral resolution of *ca* 1 cm^−1^ at 1000 cm^−1^.

### Synchrotron X-ray measurements

XRF microscopy and μ-XANES experiments were carried out at the Sub-micron Resolution Spectroscopy (SRX) beamline (5-ID) of the National Synchrotron Light Source II (NSLS-II). A horizontal mirror focuses the synchrotron beam from an undulator into a secondary source point, with a Si (111) monochromator selecting the X-ray energy. A set of fixed-curvature Kirkpatrick-Baez mirrors in the end station reimages that source onto the area of interest in the sample with ~1 μm beam spot size^30,31^.

Mapping XRF measurements on the cross-section were done at two different energies: 6.5 keV and 14 keV. The lower energy (6.5 keV) is above and near the Pb M edge and Sn L edge, which provides sensitivity to both elements. The higher energy (14 keV) is above and near the Pb-L_3_ edge, which was the target for the XANES experiment at SRX. Spectra fitting was performed using PyXRF (developed in-house at NSLS-II). Elemental maps were generated by taking the integral of the fitted fluorescence signal corresponding to each element, after normalization by the incident beam intensity. Statistical analysis of the elemental distribution correlation, including probability density distribution and linear regression, was conducted in a Python environment, using codes written by the authors.

XANES measurements were performed on both the cross-section sample and the following standard powder samples: Pb white (2PbCO_3_·Pb(OH)_2_), PbSn yellow type I (Pb_2_SnO_4_), red Pb (Pb_3_O_4_), Pb azelate, and Pb palmitate. The incident X-ray energy was scanned across the Pb L_3_ edge by stepping the monochromator’s Bragg angle and the undulator’s gap. XANES spectra in both fluorescence and transmission modes were collected for the standards, whereas XANES spectra in fluorescence mode were collected on the cross-section sample. Analyses of the XANES spectra, including normalization, background subtraction, and linear combination fitting, were conducted using the Athena freeware package.

## Results and Discussions

### Light microscopy and Raman analysis

**Figure 1:**
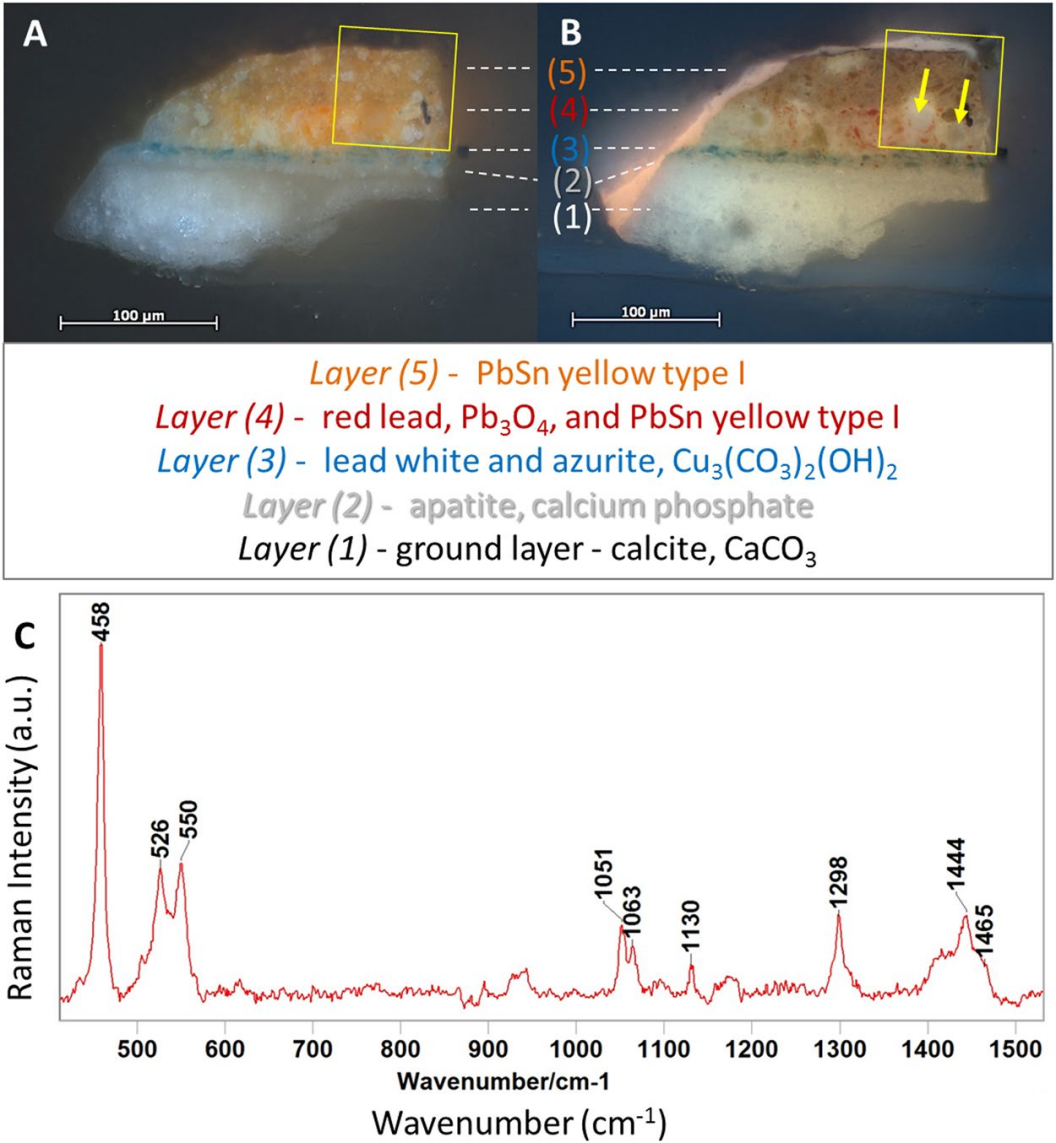
Photomicrographs of a paint cross-section removed from the original frame in *The Crucifixion* (MMA #37.92a), by Jan Van Eyck, taken with visible (**A**) and UV illuminations **(B**), respectively. The arrows indicate the locations of two lead soap aggregates, and the square in each image indicates the region chosen for synchrotron X-ray analysis. The layers 1–5 are labeled to indicate their different compositions, as determined Raman spectroscopy. (**C**) Raman spectrum representative of those acquired in the protrusions in the sample showing the presence of soaps, Pb palmitate and/or stearate (bands above 800 cm^−1^)^7^, PbSn yellow type I (features below 600 cm^−1^), and particles of Pb white (main band at ca. 1050 cm^−1^)^37^.

The examination of the paint cross-section removed from the frame of *The Crucifixion* with a light microscope suggested soap formation. Photomicrographs of this sample, taken with visible and UV light illuminations, are shown in Fig. 1A and B, respectively. Raman spectroscopic analysis indicated that the composition of the different layers in this sample, from the bottom up, are as follows: ground layer (layer 1) is calcite, CaCO_3_; the thin layer over ground, right below the blue layer (layer 2) is apatite, calcium phosphate; the blue layer (layer 3) is lead white and azurite, Cu_3_(CO_3_)_2_(OH)_2_; the orange layer (layer 4) is red Pb, Pb_3_O_4_, and PbSn yellow type I; and the yellow layer on top (layer 5) is PbSn yellow type I. Particles of the Pb white pigment were also detected in layers 4 and 5.

Pb soaps were identified by Raman spectroscopy (Fig. 1C) via their characteristic bands^7^ in the aggregates, approximately 10–20 microns in diameter that are visible in layer 4 (indicated by arrows in Fig. 1B). The presence of red Pb identified by Raman spectroscopy in the saponified layer 4 is most likely a product of the saponification process, rather than being deliberately added by the artist or being an impurity. It has been shown that Pb_3_O_4_ may be present as an impurity in PbSn yellow type I commercial pigment samples^24^, but it is also known that this compound forms inside Pb soap protrusions^32^. A similar sample removed from the frame of *The Last Judgment*, a painting that M. Ainsworth and S. Scully demonstrated to have most likely been made at the same time and with similar materials^27^, has a single yellow layer containing PbSn yellow type I, similar to layer 5 in Fig. 1A and B, but no red Pb or any other evidence of saponification (image not shown).

**Figure 2:**
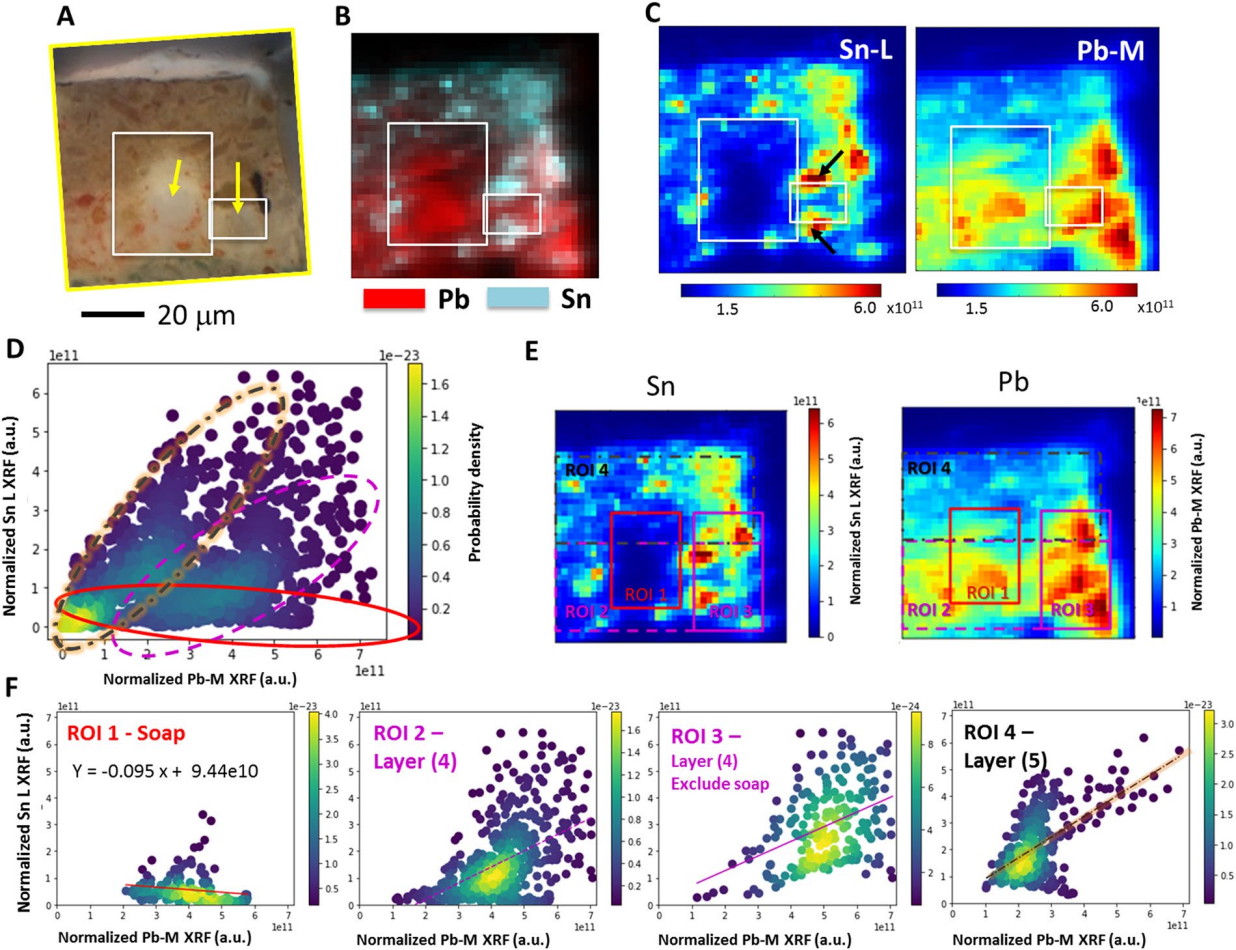
(**A**) Detail of the photomicrograph shown in Fig. 1B, where the arrows indicate the locations of Pb soap aggregates. (**B** and **C**) XRF maps of the same area collected with 6.5 keV X-ray radiation. In these images, the white rectangles highlight the areas where Sn is depleted due to saponification. The black arrows in Figure C indicate areas where there is a concentration of Sn around a region where Sn is depleted. Elemental distribution correlation between the Sn-L and Pb-M plotted as probability density distributions from the full 80 × 80 μm^2^ scanned area is shown in (**D**). Regions of Interest (ROIs) selected for the elemental distribution correlation analysis in the sample cross-section, with results shown in (**F**) - from left to right - soap region, full layer 4, layer 4 excluding the soap region, and layer 5.

The saponification observed at the bottom of the original PbSn yellow (layer 4) in the paint cross-section shown in Figs 1 and 2A may be explained by a larger supply of free fatty acids in that region. Similar partial formation of soaps in paint layers has been previously reported and ascribed to the migration of fatty acids from medium-rich paint layers above or beneath the saponified paint layer^5,33^. To characterize these soap aggregates further, the area indicated by a yellow square in Fig. 1A and B was analyzed by synchrotron XRF microscopy and XANES spectroscopy.

### X-ray fluorescence microscopy

XRF analyses at 6.5 keV were carried out in the area indicated by yellow squares in Fig. 1A and B, and presented again in Fig. 2A, to determine the elemental distribution of Sn and Pb. The corresponding XRF maps are shown in Fig. 2B, where it can be observed that Sn is depleted in the saponified area, indicated by white rectangles in this figure. Figure 2C shows the Sn-L and Pb-M XRF maps, where the Sn depletion can be clearly identified by the low intensity of the Sn XRF signal from the Pb soap regions. In this figure it can also be observed that Sn concentrates in areas surrounding the soap aggregate (indicated by black arrows in Fig. 2C). It has been shown that SnO_2_ may be present in commercial PbSn yellow pigments and that it also forms as a result of the saponification reaction of PbSn yellow with palmitic acid^24^. The Pb-Sn segregation observed is consistent with analysis conducted by Boon *et al*. using SIMS, for which a PbSn yellow type I paint sample taken from a triptych executed by an unknown Northern European artist was studied^12,34^. Similar analyses of samples created in the laboratory and aged under controlled conditions may demonstrate whether the concentration of Sn around the soap aggregates results from saponification or is due to an unreacted impurity in the artistic pigment that is being ‘pushed’ when the aggregate is formed.

The segregation Pb and Sn, observed qualitatively first, was quantified by plotting the Sn-L vs. Pb-M signal for each data point (pixel) over the entire 80 × 80 *μm* ^2^ scanned area as scattering plots (probability density distribution plots), shown in Fig. 2D. A correlation plot visualizes and quantifies the co-localization (or lack thereof) of two elements in a sample. If we consider just two elements in a sample with a homogenous composition, the ratio between these two elements will remain constant, resulting in a linear distribution with a positive slope and a positive correlation coefficient between these two elements. In a sample with a completely random elemental distribution, no correlation will be observed and the correlation coefficient will be zero. In a sample where two elements do not co-localize, a negative slope is expected in the scattering plot, along with a negative correlation coefficient.

Figure 2D shows the elemental distribution correlation between the Sn-L and Pb-M of the full image datasets (as shown in Fig. 2C). Three clusters of distribution in Fig. 2D can be identified, highlighted by three oval shapes with different colors. The different regions of interest (ROIs) selected in Fig. 2C, including the saponified regions in the layers 4 and 5 as labeled in Fig. 1A and B, are presented in Fig. 2E. In Fig. 2D, the red-solid oval highlights the contribution from the saponified region (ROI 1), shown individually in Fig. 2F (most left panel). A linear regression analysis confirmed a negative correlation, with only small amounts of Sn in this region. The pink dashed oval and the black dashed-dot oval (with orange shade for the sake of visibility) highlight the contribution from the layers 4 (ROI 2&3) and 5 (ROI4), respectively, as shown individually in the remaining three panels of Fig. 2F. The slope (Sn-to-Pb ratio) is larger in ROI 4, because layer 5 contains mainly PbSn yellow, while in layer 4 red Pb (Pb_3_O_4_) and PbSn yellow type I are present in addition to the soaps. Table 1 lists, for all four regions of interest, the fitted slopes, intercepts, correlation coefficients and two-sided p-values for a statistical hypothesis test, whose null hypothesis is that the slope is zero. For layer 4, the elemental correlations including and excluding the soap contribution were computed, both yielding positive correlations. In the next section, we will discuss the chemical composition of the different regions where elemental segregation was identified.

**Table 1:** Linear regression analysis on the elemental correlation.

Region	Slope	Intercept (1e11)	Correlation coefficient	P-value
Full image	0.35	0.42	0.49	1.16e-98
ROI 1 – Soaps	−0.095	0.94	−0.16	0.036
ROI 2 – Layer 4	0.60	0.10	0.48	3.06e-31
ROI 3 – Layer 4, excluding the soaps	0.55	0.17	0.43	2.07e-10
ROI 4 – Layer 5	0.77	1.33	0.58	1.26e-37

### XANES spectra of Pb-containing pigments and soap standards

**Figure 3:**
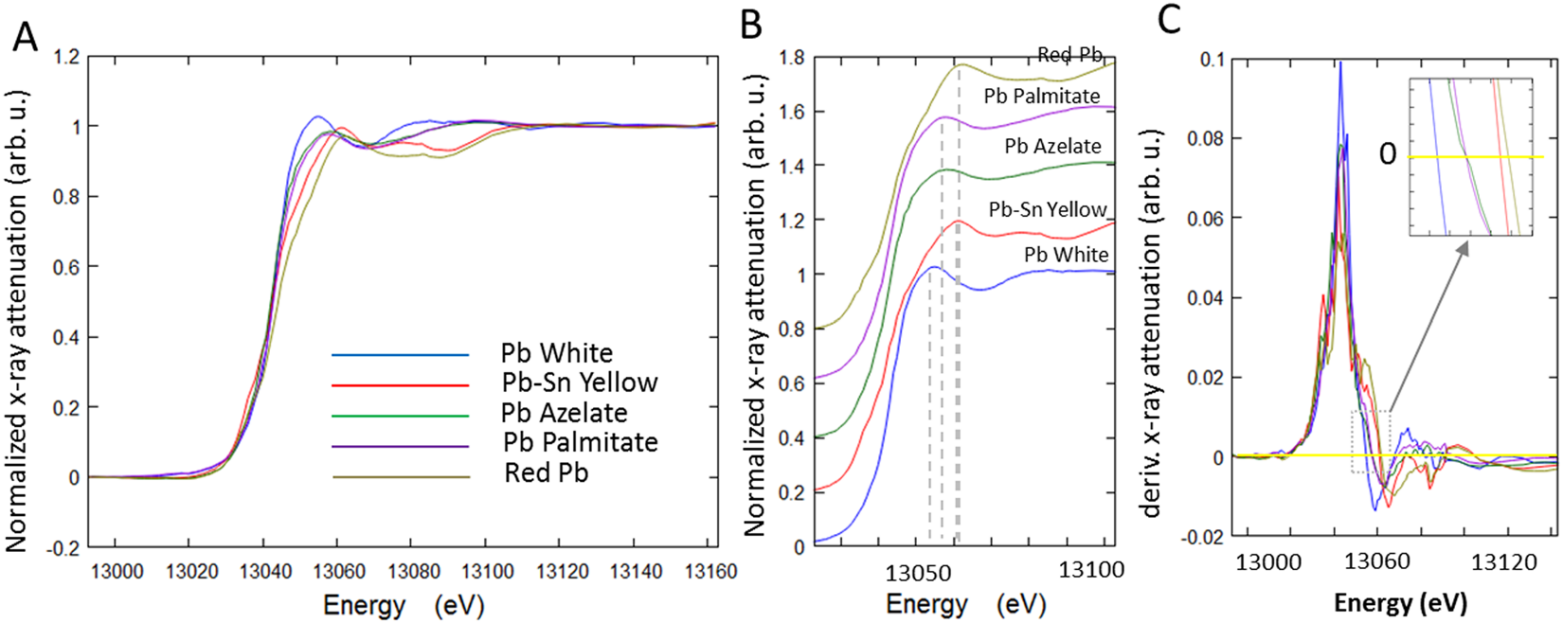
XANES spectra of Pb white, PbSn yellow type I, Pb azelate, Pb palmitate, and red Pb, collected in transmission mode around the Pb L_3_ edge. (**A**) Full XANES spectra; (**B**) energy region of interest, shown in a stacked view; and (**C**) first derivatives of the XANES spectra.

Soap inclusions in paintings dating from the 14^th^ to the 18^th^ century, from a broad range of geographical locations and studied by GC-MS and FTIR, have been reported to contain lead carboxylates of the stable saturated C_16_ and C_18_ straight-chain monocarboxylic fatty acids (palmitic and stearic acid), and little to no Pb azelate, depending on the sample^1,3,11^. Because Pb palmitate and Pb stearate have similar environments around the Pb carboxylate polar head group^23^ and are expected to have similar mobility within the paint films, XANES spectra of only Pb palmitate and azelate standards were recorded. The pigments lead white, PbSn yellow type I and red Pb can be differentiated from the soaps by their XANES spectra. Figure 3A shows the full XANES spectra of these five compounds, collected at SRX in the transmission mode. There are noticeable differences in post-edge features, as shown in the selected energy region of interest in the stacked plot (Fig. 3B). The pigments exhibit shifts in the white-line positions, which are also different from the white line positions of Pb azelate and Pb palmitate. However, the two Pb soaps cannot be differentiated by their XANES spectra, owing to the similar chemical environments of Pb in both compounds that result in similar fingerprints at this X-ray energy. To locate the white line positions more precisely, the first derivatives of the spectra were taken (Fig. 3C). The insert shows the positions where the derivatives are equal to zero at the post-edge position. The white lines were determined to be: 13054.7 eV for lead white, 13057.7 eV for the Pb soaps, 13061.0 eV for PbSn yellow type I, and 13061.7 eV for red Pb.

The results obtained for the powder samples collected in the X-ray fluorescence mode are included with the supplementary materials (Figure S1). The general trends are consistent in these two modes, however the precise white line locations are slightly different. The derivatives show the following white line positions in the XRF mode spectra: 13054.3 ± 1.0 eV for Pb white, 13055.5 ± 1.0 eV for Pb azelate, 13056.2 ± 1.0 eV for Pb palmitate, and 13060.1 ± 1.0 eV for PbSn yellow type I, and 13060.7 ± 1.0 eV for red Pb. The Pb white and Pb soap compounds are not easily differentiated in the XANES collected in the X-ray fluorescence mode, which may be attributed mainly to potential self-absorption effects. PbSn yellow type I can be clearly differentiated from the Pb soaps, so the measurements on the standard powders indicated that it is possible to apply this technique to paint samples with soap formation, such as the one removed from a 15^th^ century painting discussed in this paper. Future work to evaluate if other types of X-ray spectroscopy measurements, such as extended X-ray absorption fine structure (EXAFS) and X-ray photoelectron spectroscopy (XPS), may be used to differentiate among the different Pb soaps would be potentially beneficial.

### XANES analysis of a paint sample cross-section

**Figure 4:**
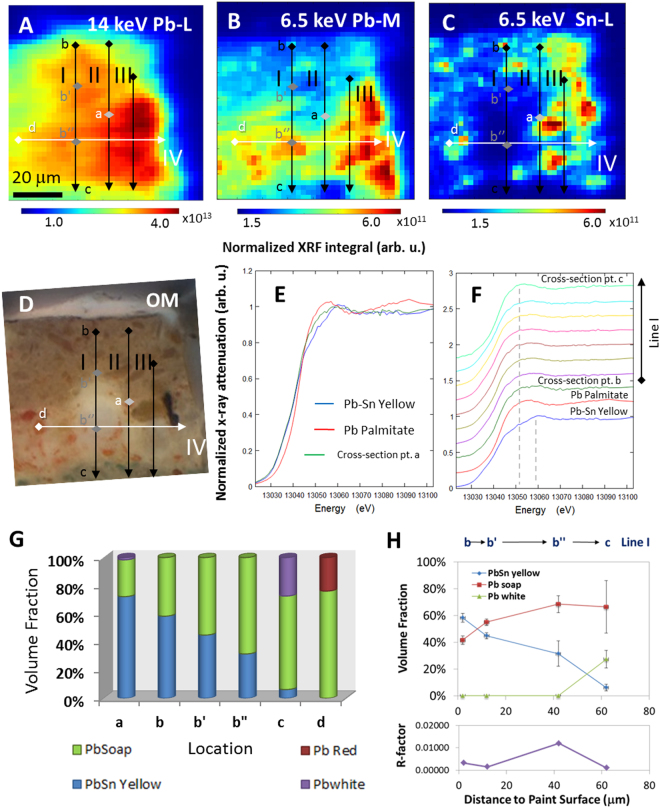
The (**A**) XRF map of Pb (Pb-L) collected with an X-ray energy of 14 keV; (**B**,**C**) XRF maps of Pb (Pb-M) and Sn (Sn-L) collected with an X-ray energy of 6.5 keV; and (**D**) photomicrograph obtained with an optical microscope, shown again to indicate the corresponding locations where the XANES spectra were collected, along lines I, II, III, and IV at Pb-L_3_ edge. (**E**) XANES spectrum acquired in the point ‘a’ in the sample cross-section compared with those obtained in the standard samples. (**F**) XANES scans measured along line I compared to spectra obtained in the standard samples. (**G**) Analysis of the chemical composition in selected locations; (H) chemical composition vs. distance to the paint surface, along line I on points b, b′, b″, and c, based on the results of the linear combination fitting with R-factor.

Chemical analyses by μ-XANES spectroscopy were conducted on the paint cross-section sample and the results were compared with those obtained on the standards. XRF map of the saponified region was first collected at 14 keV, well above the Pb-L_3_ edge at 13035 eV (Fig. 4A). The Pb distribution maps obtained are different from the Pb maps collected with the lower energy (6.5 keV), shown in Fig. 2C for the Pb-M emissions. This is due to different X-ray penetration depths at these two energies. In the supplementary material, calculations at 6.5 eV show that, for a 10-micron-thick PbSn yellow paint layer, less than 2% of the signal penetrates the sample (Figure S2). Considering self-absorption, fluorescence yield rate, and higher absorption from the air at this energy, the XRF detector is sensitive to less than 0.04% of the Pb signal. On the other hand, at 14 keV, even a 20-micron-thick PbSn yellow paint layer exhibits a 10% transmission and contributes to the detected signal, considering all the effects mentioned above.

This observation is important for the interpretation of the results of the XANES experiments carried out at the Pb L_3_ edge. Although measured with a micron-sized beam, the results reflect the chemical composition not only in the few microns below the surface of the sample, but also to depths of several tens of microns below the surface.

XANES spectra were collected along different vertical lines (lines I-III in Fig. 4A–C) and along one horizontal line (line IV in Fig. 4A–C). Data along lines I & IV was collected with a 10-micron step size, and with a 20-micron step size along lines II & III. Figure 4B–C show the XRF maps of Pb (Pb-M) and Sn (Sn-L) collected with an X-ray energy of 6.5 keV and Fig. 4D shows a photomicrograph of the paint sample taken with an optical microscope. All the XANES spectra obtained in these measurements are similar to the spectra of Pb azelate and Pb palmitate discussed above, with the exception of the one measured at the location ‘a’ (Fig. 4A–D), which is similar to spectrum of PbSn yellow or to that of a mixture of PbSn yellow and Pb soaps (Fig. 4E). In the XRF map obtained at 6.5 keV, location ‘a’ is a Sn-depleted area, implying the presence of Pb soaps on the surface; however, in the 14 keV SRF map, we are also probing below the surface and we can see that PbSn yellow is present. A set of spectra, representative of those obtained along Line I is presented in Fig. 4F. Line I goes through a soap aggregate, as seen in the photomicrograph and in the XRF images presented in Fig. 4A–D. The post-edge features are mainly consistent with the features seen for Pb azelate and Pb palmitate (Pb azelate is omitted here due to its similarity with Pb palmitate, as shown in Fig. 3). Results obtained for Lines II-IV can be found in the supplementary materials (Figure S3). Given the penetration depth of 14 keV X-rays, these results indicate that soap formation is widespread in the sample, even in areas where soap inclusions are not visible on the surface. This information complements the Raman spectra acquired on the surface in the different spots in the sample cross-section.

To quantify the chemical compositions, a linear combination fitting was performed in the Athena software^35^. All possible combinations of the standard samples (PbSn yellow, Pb azelate, Pb palmitate, Pb white, and red Pb) were examined. The R-factors from these different combinations, indication of residuals from the fitting, were compared. The full results of the analysis can be found in Table S1. Previous studies have shown that, typically both Pb palmitate and Pb stearate with variable amounts of Pb azelate are present in soap formation^1,3^. As discussed above, the XANES at Pb K-edge cannot differentiate Pb azelate, Pb palmitate, and Pb stearate, therefore, the fitting results of palmitate and azelate components are presented as ‘Pb soaps’. However, the inorganic compounds (PbSn yellow, Pb white, and red Pb) can be well differentiated from the organic ones (Pb palmitate and Pb azelate) by this technique. The results of the best fit for the selected locations (a, b, b′, b″, c, and d) are summarized in Fig. 4G, and the values are listed in Table S2.

For point a, the best fit (R-factor 0.001416) shows that a small fraction of Pb white (~1.6 vol.%) may be present, in addition to PbSn yellow and Pb soap; Raman results show that Pb white is not the main pigment component, as just a few particles of the pigment were observed by this technique. However, the next best fit, containing PbSn yellow and Pb soap only, without Pb white, statistically also has an R-factor of 0.001417, with the same level of statistical significance. Along line I, from the paint surface to the internal region (from point b, b′, b″ to c), the further away from the paint surface, the proportion of Pb organic compounds becomes higher and the PbSn yellow vol. % becomes smaller. This relationship is plotted in the top panel in Fig. 4H, with the R-factors shown in the bottom panel in the same figure.

It is worth noting that in point c, approximately 27.3 vol. % of Pb white was found, which is consistent with the results shown in Fig. 2 – Pb white is present in the blue-color layer 3. Furthermore, red Pb was observed in point d (~23.8 vol. %); this location coincides with the red-color feature identified in the photomicrographs. Further analysis, perhaps with Pb M-edge XANES or other techniques, is required to confidently distinguish Pb azelate and Pb palmitate.

Micro-beam and spatially resolved XANES allows one to perform analyses in various locations and, therefore, to better understand the heterogeneous nature of the soap formation process. As mentioned in the Introduction, possible factors affecting soap formation in oil paintings are a relatively large supply of fatty acids from the same paint layer or from medium-rich layers above or beneath^5^, and/or exposure to moisture either from the environment or due to conservation interventions with aqueous solutions. In the case of our sample, soap formation is more prominent deep into the paint, closer to the ground layer. This result suggests that moisture absorbed by the wood support and/or a larger supply of fatty acids from the adjacent layers may have triggered the reactions. The capabilities of micro-beam and spatially resolved XANES on model samples prepared in the laboratory with soaps grown under controlled conditions, along with simulations, are promising to shed light into the factors affecting this pervasive deterioration process.

## Conclusions

The spatially-resolved spectroscopic imaging capabilities of the SRX beamline of NSLS-II were used to study metal soap formation in a microsample removed from a 15th century painting to obtain information on the correlation among elemental segregation, chemical states and types of chemical compounds formed. In the XRF microscopy analysis of the microsample cross-section, Pb was found to concentrate in aggregates approximately 10 to 20 microns in diameter; a depletion in the amount of Sn was observed within the aggregates while Sn appeared to concentrate in some surrounding areas. The Pb and Sn segregation was quantified by statistical analysis of the elemental distribution correlation, including the probability density distribution and the linear regression.

The results also show that Pb white, PbSn yellow type I, and Pb soaps (of azelaic and palmitic acids) can be differentiated by Pb L_3_ XANES. The XANES spectra acquired in the paint cross-section show the presence of Pb soaps as the main component in the aggregates and of some unreacted PbSn yellow in these areas, even if this pigment is not visible in the photomicrographs taken with visible or UV illuminations. It should be stressed that the degradation of the PbSn yellow type I pigment, in addition to potentially changing the surface texture of the paint and/or its transparency, may lead to important color changes, not only due to the degradation of the yellow pigment but also due to the formation of red Pb. To quantify the chemical compositions, a linear combination fitting was performed at multiple locations inside, near, and outside the saponified regions of the sample, and it was found that soap formation is more prominent deep into the paint, closer to the ground layer.

The relatively large protrusions in the sample analyzed reflect an advanced stage of the deterioration, however smaller crystals are expected for earlier stages and when the soaps manifest themselves by an increased transparency of the paint rather than by forming aggregates. The results presented show the potential of μ-XRF mapping and XANES to study soap formation localized in paint samples removed from naturally aged works of art and set the stage for characterizing the initial stages of the process in samples from works of art and in model paint systems.

The ability to carry out elemental and molecular analysis in the same setting is beneficial and the possibility to measure at different depths by utilizing different X-ray energies is an advantage when probing elemental and molecular heterogeneity in micro-samples. Also, since samples removed from works of art should be ideally preserved for future studies, the possibility to conduct these analyses non-invasively is crucial.

However, the limitations of these techniques should also be noted. First, while at the Pb L_3_-edge, transmission mode XANES allows one to differentiate the Pb soap compounds from the Pb pigments (Pb white, PbSn yellow and red Pb), the technique cannot be used to distinguish between Pb azelate and Pb palmitate. Furthermore, one should note that when conducting X-ray microscopy and absorption spectroscopy at different energies, for their complementary capabilities, their different X-ray penetration depths and self-absorption effects will determine the depth probed. Notably when using synchrotron techniques, the accessibility to synchrotron technique and possible beam damage to the sample shall be considered. Finally, additional analysis including techniques that can differentiate Pb azelate and Pb palmitate, as well as studies on model paint samples, will be beneficial to further understand the mechanisms of the reactions. Confocal XRF microscopy and XANES using capillary optics^36^ or tomographic techniques may also be used to provide resolution in three dimensions.

## Electronic supplementary material

Supplementary Information
