# Supplementary material for: Elemental and Molecular Segregation in Oil Paintings due to Lead Soap Degradation
Source: Sci Rep. 2017 Sep 14;7:11656. doi: 10.1038/s41598-017-11525-1 (PMC5599643; doi:10.1038/s41598-017-11525-1)
Supplement: Supplementary file 1 — Supplementary Information [file 41598_2017_11525_MOESM1_ESM.pdf]

Elemental and Molecular Segregation in Oil Paintings due to Lead Soap Degradation.

Supplementary Materials

Yu-chen Karen Chen-Wiegart[1,2*], Jaclyn Catalano[3], Garth Williams[1], Anna Murphy[4], Yao Yao[4], Nicholas Zumbulyadis[5], Silvia A. Centeno[6**], Cecil Dybowski[4], Juergen Thieme[1]

[1] Department of Materials Science and Chemical Engineering, Stony Brook University, New York

[2] National Synchrotron Light Source II, Brookhaven National Laboratory

[3] Department of Chemistry and Biochemistry, Montclair State University, New Jersey

[4] Department of Chemistry and Biochemistry, University of Delaware, Newark, Delaware.

[5] Independent Researcher, Rochester, New York

[6] Department of Scientific Research, The Metropolitan Museum of Art, New York

Corresponding authors: *Karen.Chen-Wiegart@stonybrook.edu, **silvia.centeno@metmuseum.org

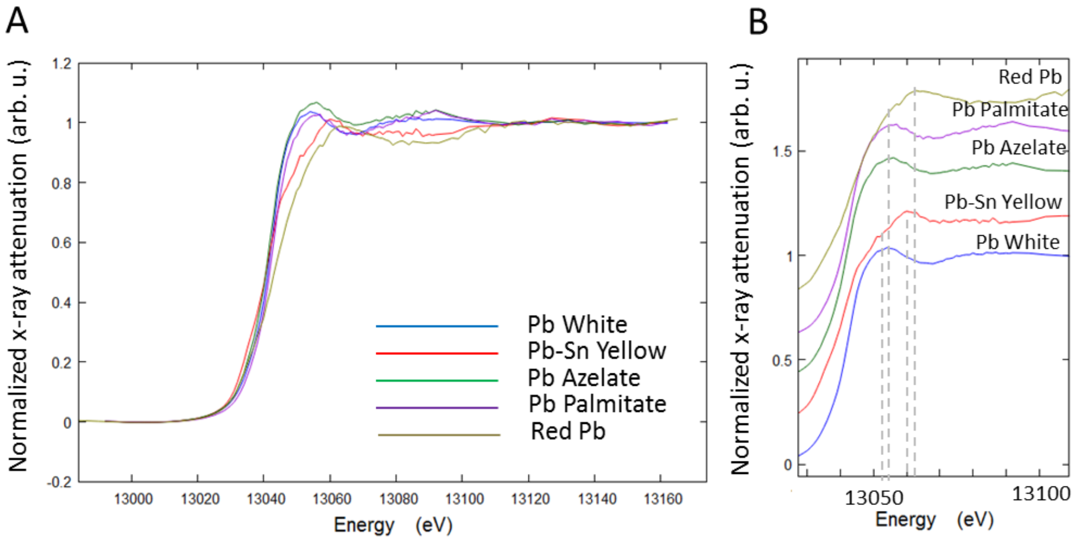

Figure S1. XANES spectra of Pb pigments and soap standards, collected in fluorescence mode around the Pb L_3 edge. (A) Full XANES spectra, (B) selected energy region of interest with stacked view.

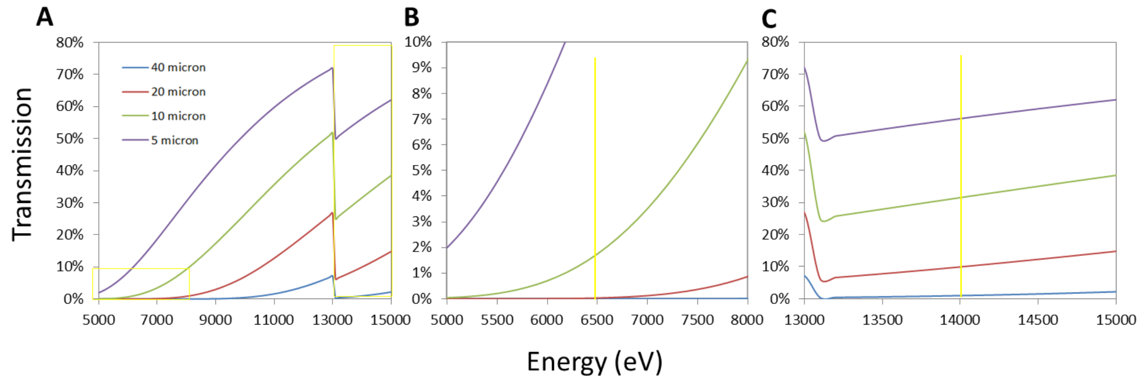

Figure S2. X-ray transmission of the PbSn yellow type I pigment with different thicknesses (5, 10, 20 and 40 microns) as a function of the X-ray photon energy: (A) energy range 5-15 keV, (B) 6.5 keV, and (C) 14 keV.

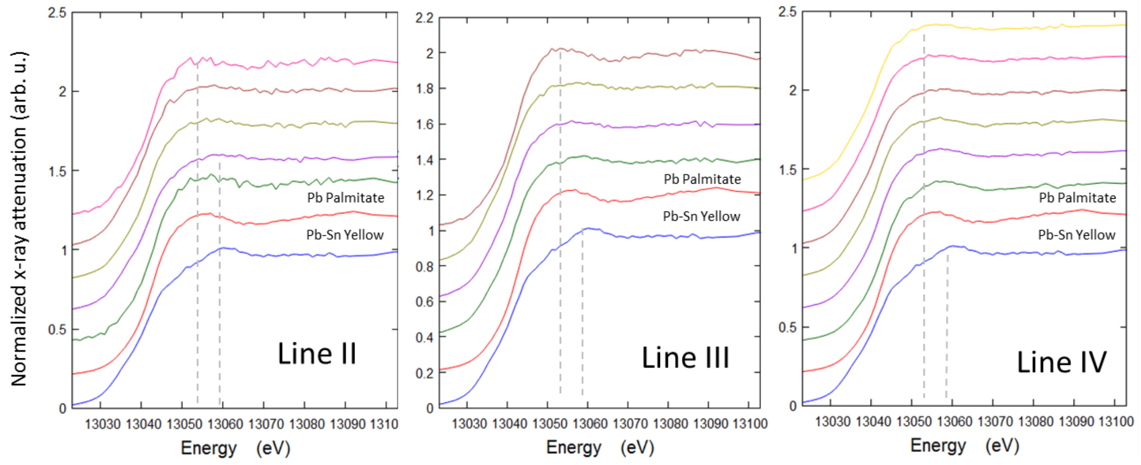

Figure S3. μ-XANES spectra acquired in the sample cross-section. The positions where the spectra were measured are indicated in Figure 4B.

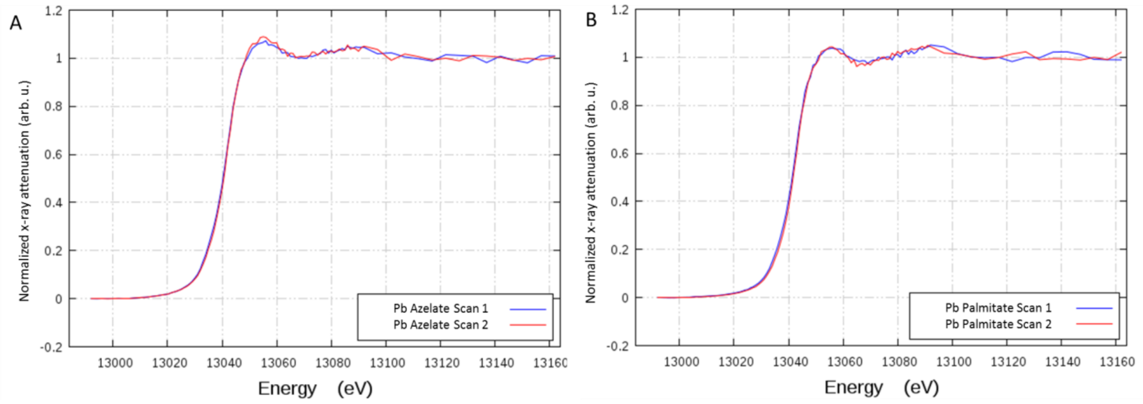

Figure S4. XANES spectra acquired consecutively in the same spot in Pb soap compounds, showing that no radiation damage is visible in the spectra.

Table S1. XANES linear combination fitting results: all combination tested, ranked by R-factor. Tests constraints: PbSn yellow must be present, weight should be between 0 and 1.

Point a

					PbSn Yellow		PbAzelate		Pb Palmitate		Pb White		Red Pb	
rfactor	chinu	chisqr	nvarys	scaleby	weight	error	weight	error	weight	error	weight	error	weight	error
0.001416	0.0001606	0.01189	2	1	0.722413	0.097624	0.261328	0.057983			0.016259	0.068027		
0.001417	0.0001586	0.0119	1	1	0.725764	0.037589	0.274236	0.020973						
0.00142	0.0001633	0.01192	3	1	0.73228	0.058091	0.250978	0.082676			0.016742	0.12955	7.00058E-09	0.039462
0.00144	0.0001634	0.01209	2	1	0.702545	0.06872	0.297455	0.035624	1.571E-08	0.043735				
0.001447	0.0001641	0.01215	2	1	0.752102	0.05848	0.247898	0.028214					9.74451E-09	0.032912
0.001469	0.0001713	0.01233	4	1	0.702504	0.221714	0.168212	0.097491	2.08815E-09	0.133853	0.129283	0.125836	1.7761E-08	0.074292
0.001804	0.000202	0.01515	1	1	0.698178	0.041762					0.301822	0.027767		
0.001815	0.0002059	0.01524	2	1	0.716472	0.109774			4.22811E-09	0.07412	0.283528	0.070824		
0.001832	0.0002079	0.01538	2	1	0.727942	0.059452					0.272058	0.031663	1.02822E-08	0.031484
0.003292	0.0003686	0.02764	1	1	0.781982	0.039251			0.218017982	0.223983				
0.003298	0.0003742	0.02769	2	1	0.795962	0.067605			0.204038231	0.039903			1.30268E-08	0.037915
0.004649	0.0005202	0.03901	1	1	1	0.054183							1.85661E-10	0.044303

Point b:

					PbSn Yellow		PbAzelate		Pb Palmitate		Pb White		Red Pb	
rfactor	chinu	chisqr	nvarys	scaleby	weight	error	weight	error	weight	error	weight	error	weight	error
0.003219	0.000377	0.02863	1	1	0.583558	0.032315	0.416442	0.032315						
0.003219	0.000382	0.02863	2	1	0.586052	0.084839	0.413948	0.103742					2.90335E-08	0.050204
0.003223	0.000382	0.02866	2	1	0.593474	0.039077	0.406526	0.11655			7.93966E-08	0.104941		
0.003499	0.000421	0.03112	3	1	0.546492	0.237282	0.292198	0.101086	6.48E-09	0.114155	0.161309438	0.179109		
0.003591	0.000432	0.03194	3	1	0.549706	0.202793	0.25288	0.134412			0.197414286	0.134046	4.76499E-09	0.064164
0.003813	0.000465	0.03392	4	1	0.580728	0.365195	0.224091	0.160484	0.025734	0.220517	0.169447179	0.207458	1.77516E-08	0.122362
0.004159	0.0005	0.03699	3	1	0.595069	0.254101	0.29885	0.163736	0.106081	0.153356			1.38015E-08	0.115182
0.004643	0.000543	0.04129	1	1	0.563661	0.045527					0.436339394	0.045527		
0.004714	0.000559	0.04192	2	1	0.612901	0.073222					0.387098818	0.051914	2.13793E-08	0.051638
0.005385	0.000647	0.04788	3	1	0.620555	0.34537			0.078544	0.226186	0.300901198	0.240831	2.35735E-10	0.095645
0.008132	0.000951	0.0723	1	1	0.719104	0.063043			0.280896	0.063043				
0.00827	0.000981	0.07354	2	1	0.64723	0.094839			0.35277	0.112974			9.29183E-09	0.06139
0.010259	0.0012	0.09119	1	1	1	0							0	0.079159

Point b’:

					PbSn Yellow		PbAzelate		Pb Palmitate		Pb White		Red Pb	
rfactor	chinu	chisqr	nvarys	scaleby	weight	error	weight	error	weight	error	weight	error	weight	error
0.001514	0.000196	0.01487	1	1	0.449049	0.02329	0.550951	0.02329						
0.001515	0.000198	0.01487	2	1	0.452842	0.028153	0.547158	0.080664			1.23E-07	0.075592		
0.001515	0.000201	0.01488	3	1	0.452528	0.064464	0.545977	0.120161			0.001494	0.091465	1.19657E-08	0.043785
0.001521	0.000199	0.01493	2	1	0.435712	0.061296	0.564288	0.071209					4.79938E-10	0.036243
0.001537	0.000201	0.0151	2	1	0.423883	0.029015	0.576117	0.056552	7.77101E-09	0.048541				
0.001667	0.000221	0.01637	3	1	0.384103	0.030688	0.501366	0.156967	1.51006E-08	0.082736	0.114531	0.129814		
0.001723	0.000229	0.01691	3	1	0.392745	0.095545	0.578196	0.161094	2.4073E-08	0.103708			0.029059015	0.077892
0.002535	0.000341	0.02488	4	1	0.43308	0.313119	0.259828	0.137466	0.095290416	0.188866	0.211802	0.177611	1.57194E-08	0.104827
0.003221	0.000416	0.03159	1	1	0.404992	0.039826					0.595008	0.039826		
0.003221	0.000421	0.03159	2	1	0.408136	0.146606			1.9105E-08	0.105999	0.591864	0.101279		
0.00323	0.000428	0.03168	3	1	0.423494	0.104226			5.00184E-08	0.183904	0.576505	0.237583	1.18739E-08	0.077768
0.003326	0.000435	0.03262	2	1	0.467785	0.070816					0.532214	0.045807	2.96941E-08	0.045548
0.007552	0.000975	0.07409	1	1	0.541577	0.063835			0.45842349	0.063835				
0.007675	0.001004	0.07528	2	1	0.612103	0.090147			0.387896798	0.065355			3.82368E-10	0.06209
0.012692	0.001636	0.12436	1	1	1	0							0	0.115166

Point b”:

					PbSn Yellow		PbAzelate		Pb White		Red Pb		Pb Red	
rfactor	chinu	chisqr	nvarys	scaleby	weight	error	weight	error	weight	error	weight	error	weight	error
0.011982	0.001476	0.11216	1	1	0.314909	0.095078	0.685091	0.063957						
0.011988	0.001496	0.11221	2	1	0.326946	0.077307	0.673054	0.230575			3.92144E-08	0.207601		
0.011989	0.001517	0.11222	3	1	0.30184	0.177035	0.69816	0.330009			1.62362E-08	0.251192	8.67368E-09	0.12028
0.012071	0.001527	0.11299	3	1	0.266915	0.080585	0.733081	0.41234	1.7181E-09	0.217421	3.36042E-06	0.340967		
0.012097	0.00151	0.11323	2	1	0.260435	0.168762	0.739565	0.20624					7.74528E-11	0.099818
0.012314	0.001558	0.11526	3	1	0.256651	0.249371	0.732786	0.420418	1.2716E-08	0.270621			0.010563	0.203296
0.014639	0.001877	0.13703	4	1	0.427992	0.735802	0.347924	0.322554	0.0018948	0.443272	0.222189827	0.416974	1.63015E-08	0.245993
0.018899	0.002328	0.17691	1	1	0.36837	0.117595					0.631630382	0.094229		
0.018944	0.002364	0.17733	2	1	0.328141	0.181786					0.671859473	0.230669	3.75933E-09	0.106221
0.019115	0.002418	0.17892	3	1	0.299345	0.247727			2.3357E-08	0.437212	0.698150599	0.535441	0.002503947	0.184861
0.027321	0.003365	0.25574	1	1	0.672005	0.13786			0.32799501	0.118558				
0.02765	0.003451	0.25882	2	1	0.558519	0.177928			0.44148072	0.242854			8.3546E-10	0.115168
0.030072	0.003704	0.28149	1	1	1	0.137612							7.02654E-12	0.118269

Point c:

					PbSn Yellow		PbAzelate		Pb Palmitate		Pb White		Red Pb	
rfactor	chinu	chisqr	nvarys	scaleby	weight	error	weight	error	weight	error	weight	error	weight	error
0.001038	0.0001472	0.01104	2	1	0.061878	0.024246	0.664653	0.196805			0.273469	0.065116		
0.001041	0.0001496	0.01107	3	1	0.072742	0.055609	0.670301	0.211293			0.256957	0.07887	5.09735E-10	0.037779
0.001067	0.0001554	0.01134	4	1	0.08749	0.087619	0.623807	0.092813	2.25166E-08	0.127549	0.288703	0.223281	2.20255E-08	0.07079
0.001122	0.0001612	0.01193	3	1	0.121119	0.026194	0.709653	0.227739	1.09322E-08	0.070649	0.169228	0.11083		
0.001283	0.0001794	0.01363	1	1	0.118206	0.022299	0.881794	0.185477						
0.001283	0.0001819	0.01364	2	1	0.122351	0.027572	0.877649	0.191817	1.4702E-08	0.046141				
0.001285	0.0001846	0.01366	3	1	0.109936	0.085818	0.890064	0.234174	2.5008E-08	0.093131			2.30072E-10	0.069963
0.001297	0.0001838	0.01378	2	1	0.138484	0.05888	0.861516	0.196431					3.37617E-09	0.034842
0.001438	0.0002038	0.01528	2	1	0.862325	0.043241			0.767928561	0.029439			-0.63025343	0.131723
0.002803	0.0004026	0.02979	3	1	0.139012	0			-0.13901239	0.046141	1	0	1.14909E-08	0
0.003024	0.0004229	0.03214	1	1	0.00029	0.040163					0.99971	0.040163		
0.003034	0.00043	0.03225	2	1	0.020431	0.149827			1.56234E-08	0.107119	0.979569	0.102354		
0.011159	0.0015607	0.11862	1	1	0.143617	0.08075			0.856383272	0.26556				
0.027673	0.0038705	0.29416	1	1	1	0.21167							9.1112E-09	0.120891

Point d:

					PbSn Yellow		PbAzelate		Pb Palmitate		Pb White		Red Pb	
rfactor	chinu	chisqr	nvarys	scaleby	weight	error	weight	error	weight	error	weight	error	weight	error
0.000765	0.000098	0.00705	3	1	5.33979E-08	0.06255	0.208843	0.072499	0.552806	0.067905			0.238351	0.129967
0.000771	0.0001	0.00711	4	1	1.92363E-08	0.070352	0.20516	0.074506	0.530289	0.102483	0.023188	0.096366	0.241364	0.18276
0.00084	0.000108	0.00774	3	1	0.079672365	0.052238			0.680543	0.092235	0.087441	0.098199	0.152343	0.15489
0.000849	0.000107	0.00782	2	1	0.116843513	0.03136			0.760435	0.021356			0.122721	0.067463
0.001274	0.000159	0.01174	1	1	0.256782051	0.025754			0.743218	0.103811				
0.001274	0.000163	0.01175	3	1	0.260174679	0.026355	2.99E-08	0.062947	0.739825	0.132496	2.1E-08	0.111544		
0.001275	0.000161	0.01175	2	1	0.262878718	0.102668			0.737121	0.065572	1.19E-08	0.062656		
0.001744	0.00022	0.01609	2	1	4.73737E-09	0.055504					0.719139	0.032604	0.280861	0.085179
0.001767	0.000226	0.0163	3	1	6.85245E-08	0.068413	0.303255	0.097342			0.348392	0.097071	0.348353	0.163371
0.002518	0.000318	0.02323	2	1	5.39795E-08	0.077471	0.607022	0.039286					0.392978	0.103233
0.004868	0.000607	0.0449	1	1	0.385347137	0.048116					0.614653	0.106097		
0.008048	0.001003	0.07422	1	1	0.560451338	0.052733	0.439549	0.090154						
0.015589	0.001942	0.14371	1	1	0.973622721	0.085618							0.026377	0.102187

Table S2. XANES linear combination fitting results: best fit results for points a, b, b', b", c and d, with volume fraction of standards, statistical error, and R-factors

Location	PbSn Yellow	error	PbAzelate	error	Pb Palmitate	error	Pbwhite	error	Pb Red	error	rfactor
a	0.722413114	0.097624	0.26132811	0.057983			0.016258775	0.068027			0.001416
b	0.583558022	0.032315	0.416441978	0.032315							0.003219
b'	0.449049185	0.02329	0.550950815	0.02329							0.001514
b"	0.314909059	0.095078	0.685090941	0.063957							0.011982
c	0.061877635	0.024246	0.664653499	0.196805			0.273468867	0.065116			0.001038
d	5.33979E-08	0.06255	0.20884347	0.072499	0.552805884	0.067905			0.238350593	0.129967	0.000765
